# Multimodal Approach in Minimizing Transient Neurological Complications Following Single Shot Brachial Plexus Block: A Prospective Observational Study

**DOI:** 10.7759/cureus.35667

**Published:** 2023-03-01

**Authors:** Parul Kaushik, Nitin Hayaran, Ishan Goel

**Affiliations:** 1 Anaesthesiology, Lady Hardinge Medical College, New Delhi, IND; 2 Radiology, Lady Hardinge Medical College, New Delhi, IND

**Keywords:** peripheral nerve stimulation, usg, interscalene block, supraclavicular block, transient neurological deficit, injection pressure monitoring, single shot brachial plexus block

## Abstract

Purpose: The aim of the study was to assess the benefit of a using multimodal approach, including injection pressure monitoring (IPM) and different techniques of nerve localization, with respect to complications following single-shot brachial plexus block (SSBPB).

Methods: In this study, 238 adults (132 males and 106 females) undergoing upper-limb surgeries under peripheral nerve block (PNB) were evaluated. Of these, 198 patients were given supraclavicular block, and 40 patients received interscalene block using either ultrasound (USG) and peripheral nerve stimulation (PNS) or PNS alone. Injection pressure monitoring was used in 216 patients.

Results: Transient neurological deficit (TND) was observed in six out of 198 patients where USG and NS were used along with IPM as opposed to 12 out of 18 patients without IPM (p<0.0001). In patients where only PNS was used, transient neurological deficit (TND) was seen in six out of 18 patients with IPM as opposed to all the patients (n=4) without IPM (p<0.02). Among the patients where injection pressure was monitored, six out of 198 patients developed TND when both USG and NS were used, compared to six out of 18 patients where only PNS was used (p<0.007).

Conclusion: Use of injection pressure monitoring along with different nerve localization techniques results in fewer transient neurological deficits.

## Introduction

Peripheral nerve block (PNB) involves administering local anesthetic (LA) next to target nerves, the success of which depends on the location of the needle tip and the spread of the drug. PNB, despite its advantages, is known to have complications, which can be acute or chronic. Peripheral nerve injury (PNI), although rare, is a possible complication following PNB. The incidence of PNI varies from 2.6 to 17% [1-5]. Fortunately, most injuries are transient and often subclinical or present as mild mononeuropathies. The incidence of transient neurological deficits (TNDs) is higher and varies between 8 and 10.1% in the immediate days following the block [6-8]. Permanent injuries are rare and account for only 0.02-0.4% [4,9,10]. A PNI may present as a sensory (anaesthesia, paraesthesia, hypoesthesia, hyperesthesia, and pain in the area supplied by the affected nerve) and/or motor deficit (paresis and even paralysis of affected muscles). The mechanism of PNI related to the use of PNB falls into one of three categories: mechanical and injection injury (traumatic), vascular (ischemic), and chemical (neurotoxic) [11].

Nerve axons are bundled as fascicles and enveloped within the perineurium, which consists of layers of tightly fitting perineurial cells that prevent diffusion of potentially toxic substances into the fascicle and also partially protect against mechanical injury. Multiple fascicles are surrounded by a permeable epineurium, which contains the fascicles as well as various amounts of interfascicular connective tissues [4]. Intrafascicular injections have been associated with the highest incidence and severity of PNI [12]. The onset of neurologic complications after regional anesthesia is a complex process and may result from an interaction of host, agent, and environmental risk factors [13], prior neurological deficit [1,2,14], direct trauma with needle or catheter [1,14], needle size and type (including gauge and bevel of the needle) [2,10,14], direct toxicity of local anesthetics and adjuvants [15], decreased neuronal blood flow [14], improper positioning (e.g., elbow flexion) [14], tourniquet pressure [14,16,17], and duration of compression [14,16,17].

Even after the advancement in the technique of nerve localization from paresthesia to peripheral nerve stimulation (PNS) to ultrasound (USG), the incidence of neurological injury related to PNB has not decreased [1,18,19]. During peripheral nerve stimulation, the distance from the nerve and the intensity of the motor response are poorly correlated. A needle might be placed intraneurally yet provoke no motor response [4]. But if the motor response is obtained at less than 0.2 mA, the needle is likely intraneural or engaged in the epineurium of the nerve [4,9,13,19-21]. Monitoring of injection pressures while giving a block can detect needle-nerve contact (NNC) [3,4,5,9]. The common practice of subjectively assessing injection pressure by hand feel is inaccurate [4,22-24]. High (more than 1034 mm Hg) initial periods of injection pressure might indicate intraneural injection and are associated with histological changes in neural architecture and persistent motor deficits [23]. Two objective methods for measuring injection pressure are the compressed air injection technique (CAIT) and the commercially available in-line pressure manometer.

Available literature does not assert the benefit of pressure monitoring and a multimodal approach in reducing the incidence of TND. The three methods: PNS/USG/pressure monitoring (PM) can help avoid intraneural injection. However, none of these as individual modalities decreases the incidence of PNI [4,20]. Though simultaneous use of these can be done in combination when appropriate [4]. The main aim of our study was to assess neurological complications following single-shot brachial plexus block (SSBPB) and how to minimize them using injection pressure monitoring and techniques of nerve localization, including USG and peripheral nerve stimulation, as part of a multimodal approach.

## Materials and methods

The present study was conducted in the Department of Anesthesiology of Lady Hardinge Medical College, New Delhi, India, during a two-year period from March 2019 to March 2021. The author agreed to abide by the ethical guidelines for biomedical research on human subjects while conducting the research project and received approval from the institutional review board (LHMC/ECHR/2019/111). The study population comprised adults from 18 to 65 years (American Society of Anesthesiologists (ASA) classes I and II) of age undergoing any upper limb surgery along with peripheral nerve block. Refusal by the patient is the exclusion criterion. After the pre-anesthetic checkup, written informed consent was taken from the patient.

**Figure 1:**
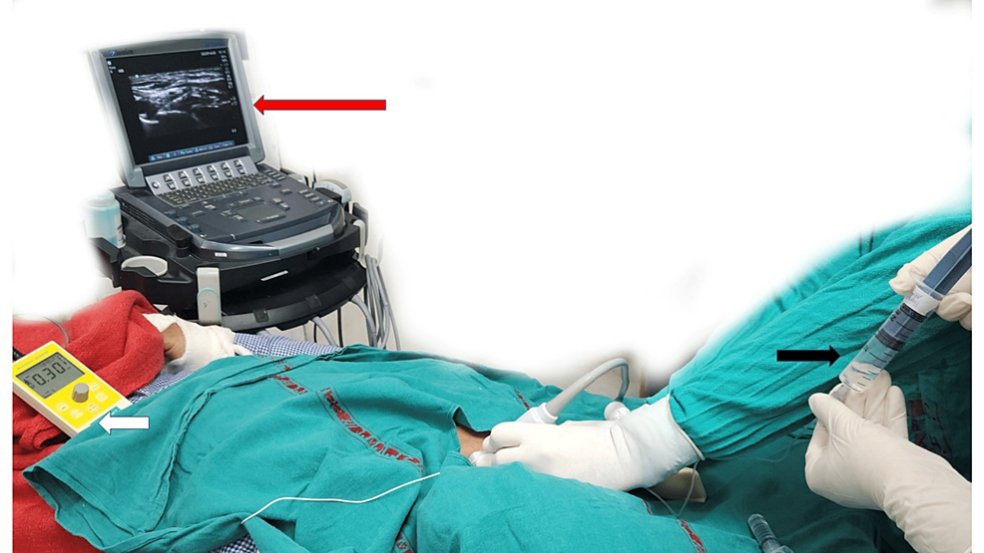
Multimodal approach for nerve localization and block administration. Red arrow: ultrasound for nerve localization, white arrow: nerve stimulator, and black arrow: compressed air injection technique.

**Figure 2:**
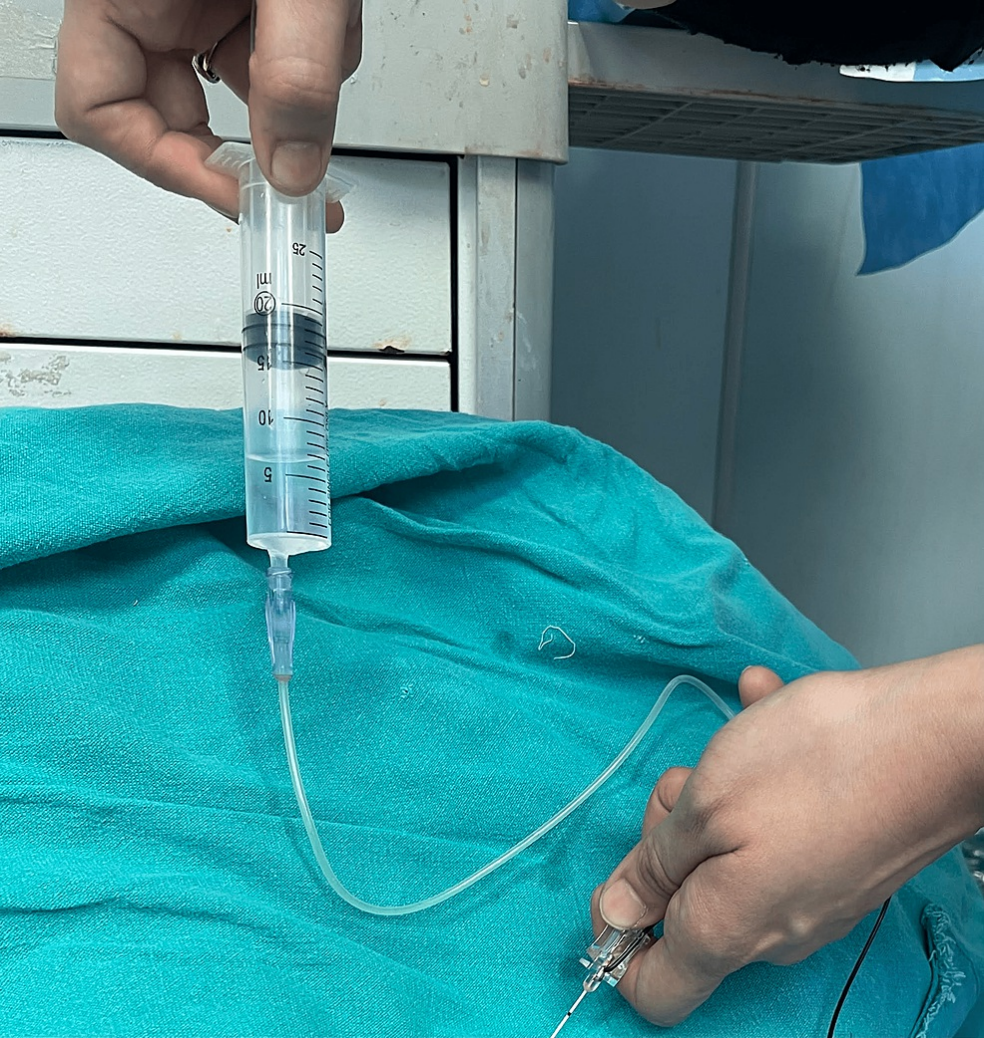
Demonstration of compressed air injection technique (CAIT). While delivering the drug, 50% of an air column (5 ml) is compressed.

Block was administered using a standard 22-gauge, 5-cm stimulating needle with nerve localization by both USG and nerve stimulation or using nerve stimulation alone (Figure 1). A needle adjustment was done in patients who had a motor response at <0.2 mA. Injection pressure monitoring was done using CAIT, using a 20-ml syringe filled with 10 ml of air above 10 ml of LA and compressing this air column to 5 ml before and during injections (Figure 2). A needle adjustment was done in patients where high pressure was encountered, i.e., more than 50% of air column compression.

Depending on the block site and the technique used by the anesthetist, the following parameters were observed: nerve localization method adopted (electrical nerve stimulation, paresthesia), local anesthetic used (bupivacaine along with lignocaine and clonidine or fentanyl as adjuvants), pressure monitoring (CAIT), and tourniquet time.

In the post-operative period, further monitoring was done for the duration of post-operative analgesia (visual analog scale (VAS)>3), sensory deficit (in dermatomal distribution) at 24 h, 48 h, 72 h, day 4, day 5, day 6, one week, one month, and three months of the post-operative period, motor deficit (according to the nerve involved) at 24 h, 48 h, 72 h, day 4, day 5, day 6, one week, one month, and three months of the post-operative period (one- and three-month follow-up either by hospital visit or via telephonic communication), reported deficit was managed as per standard treatment protocol.

A VAS>5 on emergence implied inadequate analgesia. Any sensory deficit and pain (pinprick) sensation in the dermatomal distribution of upper limb nerves were assessed 24 and 48 h after surgery. The presence of paresthesia or hypoesthesia was also documented. The motor deficit was assessed according to specific nerve-related movements of the upper limb, and it was followed up at 72 h, day 4, day 5, day 6, one week, one month, and three months. The patient was considered to have a transient neurological deficit (TND) if the symptoms continued or new symptoms developed at more than or equal to 72 h. If still some deficit was present at the end of one week then a nerve conduction velocity (NCV) study was done, and the patient was managed according to the standard treatment protocol. The frequency of neurological complications after single-shot brachial plexus block, factors associated with neurological complications and the frequency of individuals developing long-term complications were observed.

## Results

A total of 238 patients aged 18-65 years were included in the study, with a mean age of 36.45 years. About 55.5% of the patients (132/238) were males, out of which six patients (4.5%) developed TND, and 44.5% (106/238) of the patients were females, out of which 18 patients (17%) developed TND.

Out of 238 patients, 10 patients were diabetic of which 2 (20%) patients developed TND, whereas 22 (9.6%) out of the 228 non-diabetic patients developed TND.

Twenty-four patients were clinically obese (BMI>30), of which 6 (25%) patients developed neurological deficits, whereas out of the 214 non-obese patients, 18 (8.4%) developed neurological deficits. The association of sex and patient-related factors (namely, diabetes and obesity) with the development of TND was not found to be statistically significant.

Among the upper limb surgeries performed, the most common operative site was the radius, seen in 116 patients (48.7%). Other sites included shoulder, humerus, elbow, ulna, both radius and ulna, and wrist. The frequency of TND was seen in most of the operative sites, except in cases where both radius and ulna surgery were performed. Nonetheless, the association of TND with the surgical site was statistically insignificant (Table 1).

**Table 1:** Association of sensory deficit with the surgical site.

Surgical site	Sensory deficit	
	Yes	No
Shoulder (n=40)	6 (15%)	34 (85%)
Humerus (n=12)	2 (16.6%)	10 (83.3%)
Elbow (n=22)	2 (9.1%)	20 (90.9%)
Radius (n=116)	12 (10.3%)	104 (89.7%)
Radius and ulna (n=8)	0	8 (100%)
Ulna (n=38)	2 (5.3%)	36 (94.7%)
Wrist (n=2)	2 (100%)	0

Supraclavicular block was administered to 198 patients, out of which 18 patients (9.1%) developed TND, whereas 40 patients were given interscalene block, out of which six patients (15%) developed TND, with no significant difference between the two groups.

Tourniquet was applied in 132 patients; of these, in 88 patients, the duration of the tourniquet application was less than or equal to 90 minutes, and 16 of these patients (9%) developed TND. In 44 patients, the tourniquet time was more than 90 minutes, for which intermittent deflation was done, and 12 of these patients (13.6%) developed TND. The difference between the two groups was clinically insignificant.

The mean duration of analgesia (VAS<3) associated with the block was found to be 11.8 ± 4.29 h. The anatomical neural distribution of TND and the time interval of development of the neurological deficit are shown in Table 2. About 100 (42%) patients out of 238 had a sensory deficit at 24 h. In 66 (27.7%) of these patients, the deficit lasted till 48 h, whereas 24 patients (10.1%) had a neurological deficit till 72 h (considered as TND in our study). Out of these, in 6 (2.5%) patients deficit lasted till the fourth day, whereas 4 (1.5%) patients had deficit till the fifth day. Only 2 (0.8%) patients had a persistent deficit till one month for which NCV was done. One patient showed compression of the radial nerve at the mid-humerus level, subsequently, surgical exploration was done, after which the patient was relieved of the neurological symptoms. The other patient was observed to have no significant nerve compression on NCV and was managed conservatively. Repeat NCV done after one week showed similar findings, and patient symptoms gradually improved with limb physiotherapy. None of the patients had any sensory deficits after three months (Table 2).

**Table 2:** Summary of the neurological deficit with its distribution at various time intervals.

Neurological deficit		24 h	48 h	72 h	D4	D5	D6	D7	One month	Three month
Sensory		100 (42%)	66 (27.7%)	24 (10.1%)	6 (2.5%)	4 (1.5%)	2 (0.8%)	2 (0.8%)	2* (0.8%)	0
Distribution of sensory deficit	Radial	52 (52%)	32 (48.5%)	12 (50%)	6 (2.5%)	4 (1.5%)	2 (0.8%)	2 (0.8%)	2 (0.8%)	0
	Diffuse	20 (20%)	16 (24.2%)	10 (41.7%)	0	0	0	0	0	0
	Median	22 (22%)	14 (21.1%)	2 (8.3%)	0	0	0	0	0	0
	Ulnar	6 (6%)	4 (6.1%)	0	0	0	0	0	0	0
Motor		6 (2.5%)	0	0	0	0	0	0	0	0

On the basis of the above techniques, 238 patients in the study were categorized into four groups, and all the patients were monitored for the development of TND: (Table 3) group A: USG and NS with IPM (n=198), group B: NS with IPM (n=18), group C: USG and NS without IPM (n=18), and group D: NS without IPM (n=4).

**Table 3:** Association of the sensory deficit with injection pressure monitoring and nerve localization. USG: ultrasound.

Nerve localization		Nerve stimulator and USG		Nerve stimulator	
Sensory deficit		Yes	No	Yes	No
Pressure monitoring	Yes	6 (2.52%)	192 (80.67%)	6 (2.52%)	12 (5.04%)
	No	12 (5.04%)	6 (2.52%)	4 (1.68%)	0

Among groups A and C, where USG and NS were used for nerve localization, six patients developed TND in group A (where injection pressure was monitored) as opposed to 12 patients in group C (where injection pressure was not monitored), P<0.0001; significant.

Among groups B and D, where only NS was used for nerve localization, six patients developed TND in group B (where injection pressure was monitored) as opposed to all four patients in group D (where injection pressure was not monitored), P<0.02; significant.

Among groups A and B, where injection pressure was monitored, six out of 198 patients developed TND in group A (both USG and NS were used for nerve localization) as opposed to six out of 18 patients in group B (only NS was used for nerve localization), P<0.007; significant.

Among groups C and D, where no injection pressure monitoring was done, 12 out of 18 patients developed TND in group C (where USG and NS were used for nerve localization) as opposed to all four patients in group D (where only nerve stimulation was used), P>0.54; not significant.

## Discussion

TND following a PNB has reported an incidence varying from 2.6-17% [1-5], which has remained constant for decades despite the introduction of newer technology [18,19]. The incidence of TND varies widely between reports in accordance with the defined complications, duration of follow-up, type of study, and attributed cause of injury. With the increasing use of PNB, the need to assess TND becomes pivotal, as it is associated with significant morbidity, which can have physical, psychological, social, and economic consequences for the injured patient.

In our study, we included 238 ASA I and II patients aged 18-65 years, with a mean age of 36.45 years. One hundred thirty-two out of the 238 patients were males, and 53 patients were females. In our study, the patient’s age and sex had no significant correlation with the development of TND. Among the pre-existing morbidities, 10 out of 238 patients were diagnosed as diabetics with no clinical evidence of pre-existing neuropathy. With regard to the development of TND, no significant statistical correlation was noted. According to Kopp et al. [25], in diabetic patients, there is no additional procedural advantage (including nerve localization techniques or injection pressure monitoring) in preventing neurological complications. Twenty-four patients in our study were clinically obese (BMI>30), similar to Candido et al. [26] we observed no significant association of BMI with the incidence and evolution of neurologic sequelae after single-injection SSBPB.

In our study, the incidence of TND at 72 h was 10.1%, which reduced to 0.8% at seven days similar to the incidences of transient neurological symptoms reported from reported literature varying from 8-11% [5-8]. None of the patients had a persistent or new occurrence of the neurological deficit by the end of three months. The most common operative site was the radius; seen in 116 patients (48.7%). Other sites included shoulder, humerus, elbow, ulna, both radius and ulna, and wrist. There exists a wide variety in the literature with regard to the development of nerve injuries after SS brachial plexus block in upper limb surgeries; however, no single literature is available comparing all different surgical sites and the development of TND [4,27]. Nonetheless, no statistically significant difference was seen in our study among the various sites of surgical procedures.

Out of 238 patients, 198 patients were given a supraclavicular block, of which 18 patients (9.1%) developed TND, whereas 40 patients were given interscalene block, out of which six patients (15%) developed TND, with no significant difference between the two groups. The results were similar to the study of different brachial plexus block approaches by Fredrickson et al. and Borgeat et al. [8,28]. The incidence of TND in relation to tourniquet inflation in our study is similar to the study on tourniquet application by Horlocker et al [17]. The subset of patients who had a tourniquet application time of >90 minutes had a TND incidence of 13.6%, which was higher than the 9% incidence in patients with a tourniquet time of less than or equal to 90 minutes and also the overall incidence of 10.6%. In the study, USG along with PNS was used for nerve localization in 216 (90.8%) patients and PNS alone in 22 (9.2%) patients. With USG and PNS, 18 patients were observed to have TND, whereas 10 patients developed a sensory deficit with only PNS. Anesthesiologists typically rely on a subjective evaluation of what may be a high resistance to injection while performing a PNB because it is believed that a greater injection force may be associated with an intraneural injection. Claudio et al. [24] concluded that the syringe-feel method of assessing injection force is inconsistent, and objective means of monitoring injection pressure may help to quantify and document injection techniques as compared with the used syringe-feel method. Objective assessment of injection pressure monitoring in our study was done using CAIT.

We performed USG along with PNS-guided blocks in 216 patients, out of which pressure monitoring was done in 198 patients; 6 (3%) of these patients developed a transient neurological deficit. Out of the 18 patients in whom pressure monitoring was not done, 12 (66.7%) patients developed a transient neurological deficit. PNS technique without USG was used in 22 patients. Injection pressure monitoring was done for 18 of these patients, and six patients developed TND. In consequence, we observed a statistically significant difference in patients developing TND for whom IP monitoring was not done; this was unrelated to the nerve localization technique. While the literature is limited with regard to the absolute advantage of IPM, the study by Rambhia et al. [29] has recognized IPM as a potential safeguard against the development of TND and functional nerve damage. The statistically significant difference was also noted when both USG along with PNS were used with IPM as compared to the use of PNS alone with IPM. This was also emphasized in the second American Society of Regional Anesthesia and Pain Medicine (ASRA) practice advisory 4 on neurologic complications associated with regional anesthesia and pain medicine, and they interjected that although there is not enough evidence that PNS, ultrasound, or pressure monitoring can prevent TND, it is reasonable to consider using several of these modalities in combination when appropriate. An integrated approach was also advocated by Brull et al. [11] as a safety principle in regional anesthesia to reduce the risk of TND.

A relative limitation of this study was insufficient pre-existing data on multimodal approaches for nerve localization.

## Conclusions

Although rare, TND is a serious complication following nerve blocks. No single technique is known to completely prevent TND; thus, one of the goals of administering nerve blocks is mitigating their occurrence to the minimum possible. In our study, we have observed the advantage of a multimodal approach, using more than one technique of nerve localization along with injection pressure monitoring. Further studies should be done to analyze the cumulative benefits of these techniques.
